# Bone mineral density predicts survival in patients with hepatocellular carcinoma and portal vein tumor thrombosis

**DOI:** 10.1371/journal.pone.0330336

**Published:** 2025-08-22

**Authors:** Lukas Müller, Roman Kloeckner, Lorena Heim, Maximilian Moos, Felix Hahn, Fabian Stoehr, Tilman Emrich, Dirk Graafen, Jan-Peter Grunz, Daniel Pinto dos Santos, Arndt Weinmann, Friedrich Foerster, Jens Mittler, Jens Uwe Marquardt, Tobias Bäuerle, Aline Mähringer-Kunz

**Affiliations:** 1 Department of Diagnostic and Interventional Radiology, University Medical Center Mainz, Mainz, Germany; 2 Department of Radiology, University of Wisconsin, Madison, Wisconsin, United States of America; 3 Institute of Interventional Radiology, University Hospital of Schleswig-Holstein - Campus Lübeck, Lübeck, Germany; 4 Department of Diagnostic and Interventional Radiology, University Hospital Würzburg, Würzburg, Germany; 5 Department of Radiology, University Hospital of Cologne, Cologne, Germany; 6 Department of Internal Medicine I, University Medical Center Mainz, Mainz, Germany; 7 Department of General, Visceral and Transplant Surgery, University Medical Center Mainz, Mainz, Germany; 8 Department of Internal Medicine I, University Hospital of Schleswig-Holstein, Lübeck, Germany; Kaohsiung Medical University, TAIWAN

## Abstract

Hepatocellular carcinoma (HCC) is the third leading cause of cancer-related mortality, with portal vein tumor thrombosis (PVTT) being a common complication that significantly worsens prognosis. Recent studies have identified bone mineral density (BMD) as a prognostic factor in patients with HCC; however, its role in patients with PVTT remains unexplored. This retrospective study evaluated the prognostic value of BMD in 462 patients with HCC and PVTT treated between 2005 and 2020. BMD was measured via computed tomography attenuation at the first lumbar vertebra at the time of HCC diagnosis and PVTT onset, using an established threshold of 160 Hounsfield units (HU). Kaplan-Meier analysis assessed overall survival, and multivariate Cox regression adjusted for established prognostic factors. Median BMD was 136 HU (IQR: 113–160) at HCC diagnosis and 134 HU (IQR: 109–159) at PVTT onset. Patients with BMD ≥ 160 HU showed significantly longer overall survival both at HCC diagnosis (10.4 vs. 5.5 months, p < 0.001) and PVTT onset (8.5 vs. 4.7 months, p < 0.001). In multivariate analysis at both time points, BMD remained an independent predictor of survival, alongside tumor growth pattern, therapy, and Albumin-Bilirubin (ALBI) grade (alpha-fetoprotein reached significance only at time of PVTT diagnosis). These findings suggest that BMD independently predicts survival in HCC with PVTT and may enhance prognostic modeling and therapeutic decision-making.

## Introduction

Hepatocellular carcinoma (HCC) is the sixth most frequently diagnosed malignancy worldwide and the third leading cause of cancer-related death [1]. However, survival differs significantly across various tumor stages according to the Barcelona Clinic Liver Cancer (BCLC) classification [2,3]. Portal vein tumor thrombosis (PVTT) is a common complication of HCC and one of the strongest independent predictive factors for survival [4]. Due to the dismal prognosis, PVTT leads to the classification of such patients as advanced stage according to the BCLC classification (BCLC-C). This allocation directs patients to systemic therapy [3]. In recent years, several new systemic options, notably immunotherapeutic regimens, have expanded the therapeutic arsenal [5,6]. However, response rates remain moderate, and predicting responses continues to be a major challenge in the absence of reliable predictive biomarkers [7]. Therefore, identifying novel risk factors in patients with HCC, particularly those with concomitant PVTT, is crucial for optimizing therapeutic decision-making.

Osteopenia, characterized by a lower than normal bone mineral density (BMD), has recently garnered significant attention due to its emerging association with HCC. BMD has been identified as both a predictive biomarker in HCC development in the context of liver cirrhosis [8,9] and a prognostic indicator of overall survival (OS) [10–12]. This parameter may be of particular interest in such patients because HCC is a complex disease entity characterized in most cases not only by the tumor itself, but also by the presence of concurrent liver cirrhosis [13]. Liver dysfunction significantly impacts bone metabolism and is closely linked to both malnutrition and sarcopenia [14–16]. Furthermore, immunological interactions are postulated to occur between bone, the immune system, and the tumor [17,18]. Though dual-energy X-ray absorptiometry (DXA) is currently the standard for assessing BMD, attenuation values derived from routine computed tomography (CT) have been recognized as a viable alternative for determining BMD [19–21]. A significant benefit is that BMD can be assessed precisely using CT scans originally acquired for other clinical purposes, thereby eliminating the need to subject patients to further exposure to ionizing radiation [20,21]. Consequently, due to the availability of staging CT studies in patients with HCC, these scans are increasingly being utilized to determine BMD [11,22,23]. Recent findings indicate that low BMD independently correlates with early indicators of deconditioning and may precede the onset of sarcopenia [10,24]. Furthermore, low BMD has been shown to be of prognostic value in patients with HCC undergoing surgical resection [11,22,25] or liver transplantation [10,23]. Recently, we showed that BMD is an independent predictive factor for survival in patients with HCC undergoing transarterial chemoembolization [12]. However, in the literature, the role of BMD in patients with HCC and PVTT remains unknown [26]. Therefore, the primary goal of this study is to assess the prognostic significance of BMD in this patient population.

## Methods

### Study design and patient recruitment

This is a retrospective cohort study. The study protocol conforms to the ethical guidelines of the 1975 Declaration of Helsinki as reflected in a priori approval by the institution´s human research committee. This study was approved by the ethics committee of the Medical Association of Rhineland Palatinate, Mainz, Germany (permit number 15938). Due to its retrospective design, the requirement for informed consent was waived. All patient records and clinical data were de-identified before analysis and were stored and analyzed within a secure IT environment compliant with data protection regulations.

In total, 534 patients had a diagnosis of HCC and PVTT between January 2005 and December 2020. To ensure at least 12 months of follow‐up, the final evaluation date was December 2021. The inclusion criteria were as follows: a histologically confirmed HCC diagnosis or an imaging-based HCC diagnosis according to the European Association for the Study of the Liver (EASL) and American Association for the Study of Liver Diseases (AASLD) guidelines [27,28], the presence of PVTT, availability of a CT scan at the time of both HCC and PVTT diagnosis that includes the first lumbar vertebra (L1), age > 18 years, treatment between January 2005 and December 2020, no liver transplantation during follow-up, and availability of all clinical, laboratory, and imaging data. Patients with missing clinical, laboratory, or imaging data were excluded from the study.

### Data acquisition and BMD assessment

Baseline characteristics, including demographic data and liver disease status, were retrieved from the hospital information system. Laboratory parameters were obtained from the laboratory database. HCC diagnosis was established either histologically or based on imaging criteria according to EASL and AASLD guidelines [27,28]. All patients underwent contrast-enhanced CT imaging. CT scans obtained at the time of initial HCC diagnosis and during follow-up were evaluated for tumor burden (growth pattern, number, and size of lesions) as well as for PVTT status. Differentiation between bland thrombus and PVTT was achieved using established criteria [29]. Treatment-related data, including survival outcomes, were retrieved from a prospectively maintained clinical database established at our university medical center [30]. Data were accessed for research purposes between March 7, 2022, and June 27, 2022.

**Fig 1:**
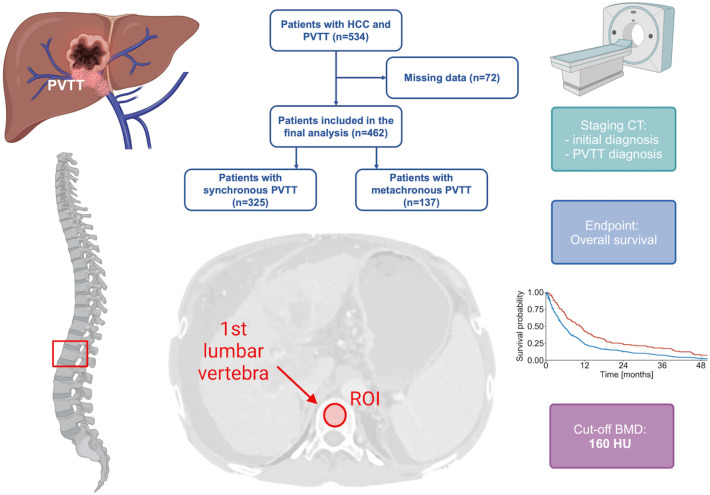
Patient selection and study methodology. This figure was created with BioRender.com.

BMD was measured at the first lumbar vertebra (L1) following previously established protocols [20,21,31]. Assessment was performed at the time of HCC diagnosis and at the time of PVTT diagnosis using the venous phase of contrast-enhanced CT. The methodology was based on the standardized approach described by Pickhardt et al. [12,20,21]^.^. A region of interest with a diameter between 10–15 mm was placed in the trabecular midvertebral core cranial to the base plate of L1 [12] (Fig 1). In cases of vertebral fractures, bone lesions, or imaging artifacts, BMD was measured in the closest adjacent vertebra cranial or caudal of L1 (n = 7, 1.5%) [12]. Based on the established cut-off of 160 Hounsfield units (HU), patients were classified into low- and high-BMD groups [10,11,21]. Additionally, the optimal cut-off value for our patient cohort was determined. Fig 1 illustrates the methodology applied.

### Statistical analysis

Statistical analyses and data visualization were conducted using R 4.4.1 (R Foundation for Statistical Computing, http://www.R-project.org; last accessed June 23, 2025). Continuous variables are expressed as medians with interquartile ranges (IQR), while categorical and binary variables are reported as absolute counts and percentages. Data normality was assessed using the Shapiro-Wilk test. Comparisons between categorical variables were conducted using Fisher’s exact test, while the Mann-Whitney U test was employed for continuous variables. Pearson correlation coefficients were calculated to evaluate associations between variables. The optimal BMD threshold for stratification was determined using an outcome-oriented cut-off selection method, commonly referred to as optimal stratification. This approach identifies the value of a continuous variable (in this case, BMD) that best separates patients into prognostically distinct groups with respect to survival. The analysis was conducted using the R packages ‘survminer’ and ‘survival’ (surv_cutpoint; https://cran.r-project.org/package=survminer and https://cran.r-project.org/package=survival, last accessed June 23, 2025), which allow for the systematic evaluation of all possible cut-off points and select the one that yields the most significant separation between survival curves, typically based on the maximization of the log-rank test statistic. This data-driven method enhances the interpretability of continuous variables in survival analysis by categorizing patients into clinically meaningful risk groups. The “survminer” and “survival” packages were also utilized for survival analyses. To assess risk stratification and the prognostic relevance of BMD, univariate and multivariate Cox proportional hazards regression models were employed, with results reported as hazard ratios (HRs) and 95% confidence intervals (CIs). Statistical significance was defined as p < 0.05 for all tests. The primary endpoints were median OS, defined as the time from initial HCC diagnosis to death or last follow-up, and from PVTT diagnosis to death or last follow-up.

## Results

### Patients

Of the 534 patients with a diagnosis of HCC and PVTT, 72 were excluded due to missing data (Fig 1). Consequently, 462 patients were included in the final analysis. Overall, 325 (70.3%) patients presented with PVTT synchronous to HCC diagnosis, whereas 137 (29.7%) developed metachronous PVTT. In patients with metachronous PVTT, the median time until development of PVTT was 251 days (IQR: 74‐558 days). Table 1 summarizes the baseline characteristics at the time of initial HCC diagnosis and at PVTT diagnosis.

**Table 1:** Baseline characteristics of the included patients.

Variable	Initial HCC diagnosis (n = 462)	PVTT diagnosis (n = 462)
Age, years, mean (SD)	65.5 (9.8)	65.9 (9.7)
Sex, n (%)		
Female	83 (18.0)	
Male	379 (82.0)	
Liver cirrhosis, n (%)		
Cirrhosis	378 (81.8)	
No cirrhosis	84 (18.2)	
BCLC stage, n (%)		
A	38 (8.2)	
B	68 (14.7)	
C	270 (58.4)	371 (80.3)
D	86 (18.7)	91 (19.7)
ALBI grade, n (%)		
1	38 (8.2)	36 (7.8)
2	284 (61.5)	280 (60.6)
3	140 (30.3)	146 (31.6)
AFP level, ng/ml, median (IQR)	266 (14–4710)	288 (17–6163)
Mean size of the largest lesion, mm (SD)	54.7 (36.3)	61.6 (43.1)
Number of lesions, median (IQR)^†^	2 (1–4)	2 (1–4)
HCC growth type, n (%)		
Diffuse	149 (32.3)	
Nodular	313 (67.7)	
Extrahepatic metastasis, n (%)		
No	372 (80.5)	358 (77.5)
Yes	90 (19.5)	104 (22.5)

Abbreviations: SD, standard deviation; BCLC, Barcelona Clinic Liver Cancer; ALBI, Albumin-Bilirubin; AFP, alpha-fetoprotein; IQR, interquartile range; HCC, hepatocellular carcinoma; PVTT, portal vein tumor thrombosis. ^†^Only patients with nodular growth type.

Additionally, Table 2 summarizes the first-line therapy after the initial HCC diagnosis and after PVTT diagnosis.

**Table 2:** First-line therapy of the included patients.

First-line therapy	after HCC diagnosis, n (%)	after PVTT diagnosis, n (%)
TACE	207 (44.8)	189 (40.9)
SIRT	12 (2.6)	13 (2.8)
MWA	3 (0.6)	0 (0.0)
Resection	47 (10.2)	34 (7.4)
Sorafenib	39 (8.4)	47 (10.2)
Epirubicin	2 (0.4)	1 (0.2)
Nivolumab	1 (0.2)	1 (0.2)
Sunitinib	1 (0.2)	1 (0.2)
Best supportive care	150 (32.5)	176 (38.1)

Abbreviations: TACE, transarterial chemoembolization; SIRT, selective internal radiation therapy; MWA, microwave ablation; HCC, hepatocellular carcinoma; PVTT, portal vein tumor thrombosis.

**Fig 2:**
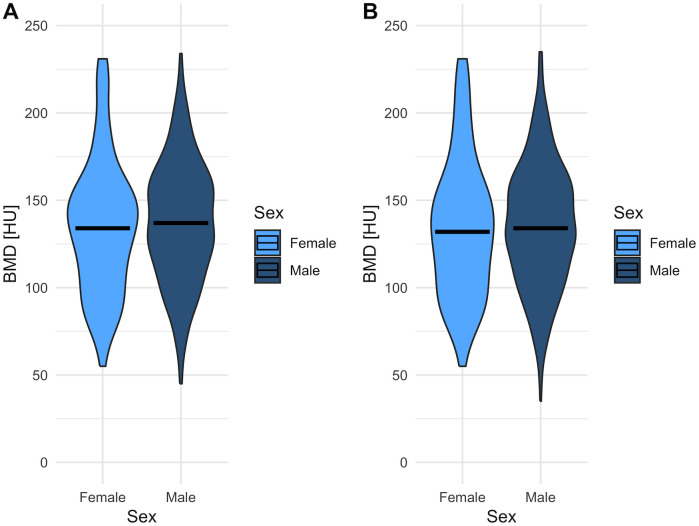
Distribution of bone mineral density (BMD) among females and males and at (A) time of first HCC diagnosis and (B) time of PVTT diagnosis.

The median BMD for the total cohort at the time of HCC diagnosis was 136 HU; at PVTT diagnosis the BMD yielded 134 HU. At neither of these timepoints significant differences in BMD could be observed between male and female patients (HCC diagnosis: females, 134 HU; males, 137 HU; p = 0.320; PVTT diagnosis: females, 132 HU; males, 134 HU; p = 0.470; Fig 2). At the time of initial diagnosis of HCC, the correlation coefficient between age and BMD was −0.29 (p < 0.01). At the time of PVTT diagnosis, the correlation coefficient between age and BMD was −0.31 (p < 0.01). Thus, we found a weak and moderate inverse correlation, respectively.

### High vs. low BMD

**Fig 3:**
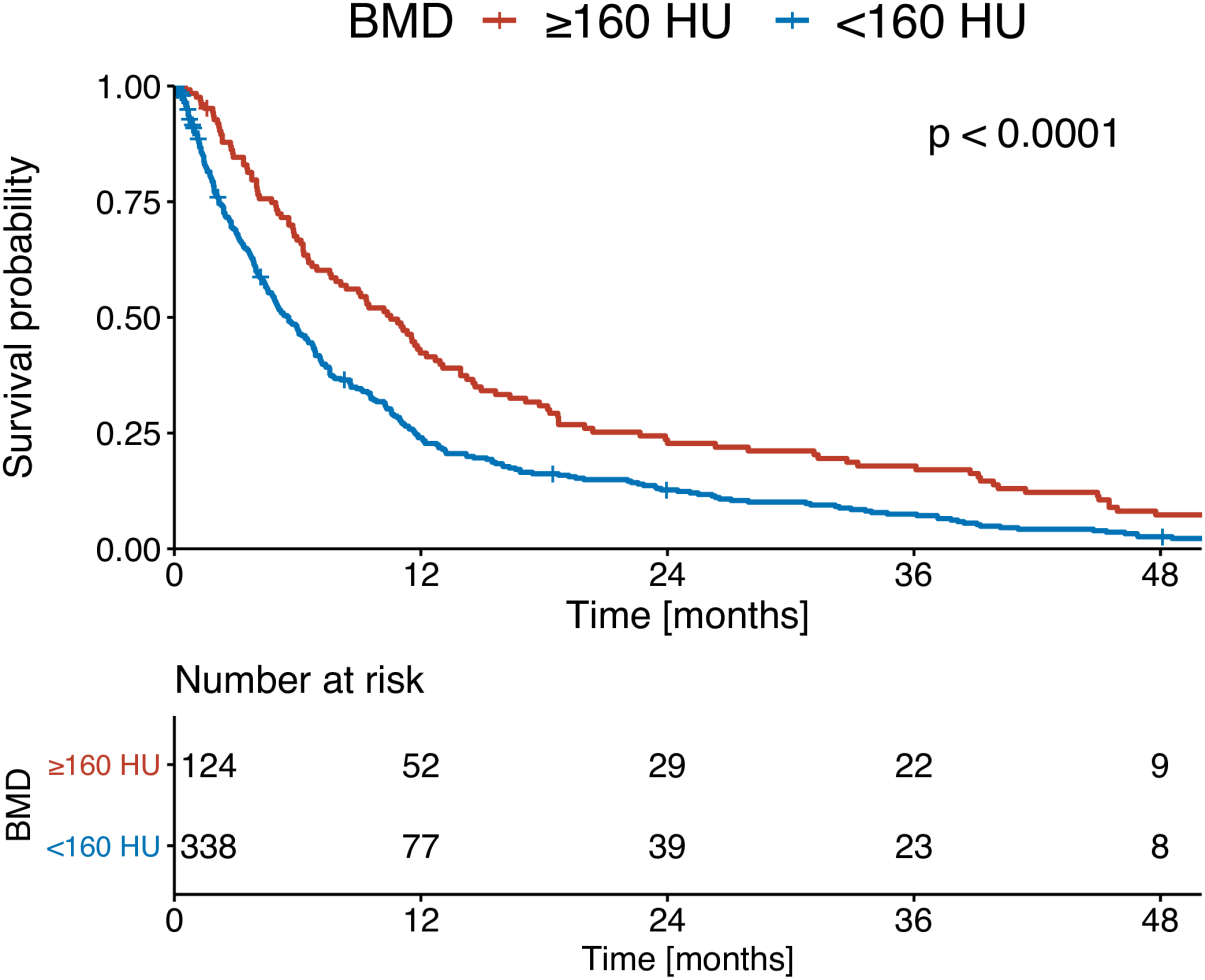
Kaplan-Meier curves of the overall survival of patients at initial diagnosis of HCC stratified according to bone mineral density (BMD).

**Fig 4:**
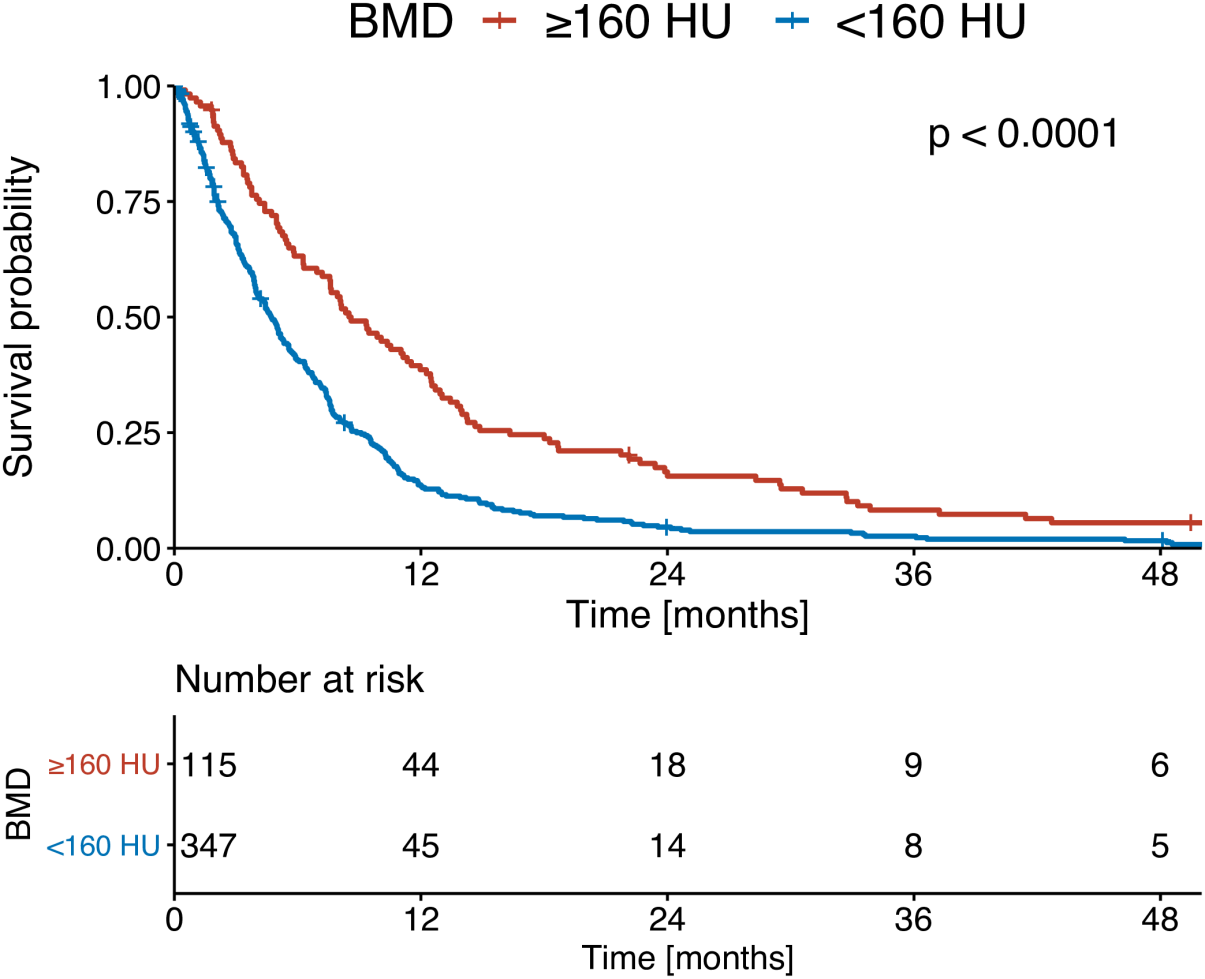
Kaplan-Meier curves of the overall survival of patients at diagnosis of PVTT stratified according to bone mineral density (BMD).

At the time of initial HCC diagnosis, patients with high BMD (≥ 160 HU) presented with a median OS of 10.4 months, whereas those with low BMD (< 160 HU) experienced a significantly shorter median OS of 5.5 months (p < 0.001; Fig 3). At PVTT diagnosis, patients with high BMD (≥ 160 HU) had a median OS of 8.5 months. Patients with low BMD (< 160 HU) had a median OS of 4.7 months (p < 0.001; Fig 4).

Beyond utilizing the established threshold of 160 HU for stratification [21], survival analysis was also conducted using the cohort-specific optimal cut-off of 159 HU.

Furthermore, a subgroup analysis was conducted to compare patients with synchronous and metachronous PVTT. In both subgroups, BMD remained a significant predictor of survival (synchronous PVTT: p < 0.001; metachronous PVTT: calculated from the date of initial HCC diagnosis, p = 0.043; calculated from the date of PVTT diagnosis, p < 0.001). A detailed breakdown of the analysis is provided in the supplemental information (S1 Fig).

### BMD as an independent prognostic factor

In the next step, a regression analysis was performed to evaluate whether BMD functions as an independent prognostic factor for OS in patients with PVTT. This analysis was performed for two different time points: at the initial diagnosis of HCC and at PVTT diagnosis. At the time of HCC diagnosis, univariate regression analysis identified tumor growth pattern, extrahepatic metastatic disease, therapy, Albumin-Bilirubin (ALBI) grade, alpha-fetoprotein (AFP), and BMD as prognostic factors for median OS. Multivariate regression analysis further confirmed that four of these variables—growth pattern, therapy, ALBI grade, and BMD—remained independent predictors of OS (Table 3). At time of PVTT diagnosis, in the univariate regression analysis, the covariates growth type, therapy, ALBI grade, and AFP level were identified as prognostic factors for median OS alongside BMD. Multivariate regression analysis confirmed that all these parameters were independent prognostic factors (Table 4).

**Table 3:** Univariate and multivariate Cox regression analyses at initial HCC diagnosis.

Analysis		Univariate			Multivariate		
Covariate		HR	95% CI	P-value	HR	95% CI	P-value
Age	≥ 67	1.0	0.9–1.3	0.680			
Sex	Female	1.2	0.9–1.5	0.150			
Growth type	Diffuse	2.8	2.3–3.5	**<0.001**	2.4	1.9–3.0	**<0.001**
Tumor node^†^	≥ 2	1.1	0.9–1.3	0.260			
Max. lesion size	> 5 cm	1.0	0.8–1.2	0.980			
EHM	Yes	1.6	1.3–2.1	**<0.001**	1.3	1.0–1.7	0.054
Therapy after initial diagnosis	BSC	3.2	2.6–3.9	**<0.001**	2.8	2.3–3.5	**<0.001**
ALBI grade	1	Reference			Reference		
	2	1.7	1.2–2.4	**0.004**	1.7	1.2–2.4	**0.004**
	3	2.6	1.8–3.7	**<0.001**	2.7	1.8–3.8	**<0.001**
AFP level	> 400 ng/mL	1.6	1.3–1.9	**<0.001**	1.2	1.0–1.5	0.074
BMD	< 160 HU	1.6	1.3–1.9	**<0.001**	1.5	1.2–1.8	**<0.001**

Abbreviations: EHM, extrahepatic metastasis; ALBI, Albumin-Bilirubin; AFP, alpha-fetoprotein; BMD, bone mineral density; BSC, best supportive care; HU, Hounsfield units; HR, hazard ratio; CI, confidence interval. ^†^Only in patients with nodular growth type.

**Table 4:** Univariate and multivariate Cox regression analyses at PVTT diagnosis.

Analysis		Univariate			Multivariate		
Covariate		HR	95% CI	P-value	HR	95% CI	P-value
Age	≥ 67	0.9	0.8–1.1	0.320			
Sex	Female	1.1	0.9–1.4	0.360			
Growth type	Diffuse	2.1	1.7–2.5	**<0.001**	1.9	1.6–2.4	**<0.001**
Tumor node^†^	≥ 2	1.1	0.9–1.4	0.110			
Max. lesion size	> 5 cm	0.8	0.7–1.0	0.062			
EHM	Yes	1.2	1.0–1.5	0.064			
Therapy after PVTT diagnosis	BSC	2.6	2.2–3.2	**<0.001**	2.6	2.1–3.2	**<0.001**
ALBI grade	1	Reference			Reference		
	2	1.3	0.9–1.8	0.157			
	3	2.1	1.4–3.0	**<0.001**	1.6	1.3–2.0	**<0.001**
AFP level	> 400 ng/mL	1.3	1.1–1.6	**0.042**	1.3	1.1–1.6	**0.004**
BMD	< 160 HU	1.8	1.5–2.3	**<0.001**	1.8	1.4–2.2	**<0.001**

Abbreviations: EHM, extrahepatic metastasis; PVTT, portal vein tumor thrombosis; ALBI, Albumin-Bilirubin; AFP, alpha-fetoprotein; BMD, bone mineral density; BSC, best supportive care; HU, Hounsfield units; HR, hazard ratio; CI, confidence interval. ^†^Only in patients with nodular growth type.

To substantiate the prognostic independence of BMD, its association with tumor burden, liver function, and AFP levels was examined. The correlation coefficients, ranging from −0.14 to 0.05 (S1 Table), demonstrated minimal association, reinforcing BMD as an independent predictor of survival.

Subsequently, a subgroup analysis was conducted to examine the influence of BMD focusing on the parameter “growth type” and “extrahepatic metastatic disease”. For both parameters, we did not observe significant differences in the median BMD at the time of initial HCC diagnosis or PVTT diagnosis, confirming the role of BMD as an independent predictive factor (S2 and S3 Figs).

## Discussion

This study evaluated the prognostic relevance of BMD in patients with HCC and PVTT. At both the initial diagnosis of HCC and PVTT onset, lower BMD was associated with significantly poorer survival compared to higher BMD. Importantly, BMD emerged as an independent predictor of overall survival in this patient cohort.

Prognostic assessment and therapy allocation for patients with HCC are determined by the BCLC classification, which includes an evaluation of the performance status (PS) of patients [2,3]. PS is a crucial parameter with high predictive relevance [32]. However, it faces several issues in clinical practice, particularly concerning its objectivity and availability. The assessment of PS will always remain subjective to a certain extent [33]. Unlike other parameters that influence categorization within the BCLC system, such as tumor size or tumor number, PS cannot be extracted from laboratory reports or imaging studies whenever needed. This results in the PS sometimes being unavailable during decision-making in tumor board meetings if the treating physician is not present. Furthermore, the PS may change over time. Consequently, novel, more objective imaging biomarkers could be useful to complement assessment of a patient’s physical condition.

As an objective imaging biomarker with broad availability, BMD has the potential to overcome the limitations of PS. The literature suggests that low BMD, as a surrogate parameter for osteopenia, is an early indicator of “physical decline”, which may even precede changes in muscle mass [10,24]. BMD has already been identified as a prognostic factor in a few studies in patients with HCC, aiding in prognostic estimation before liver transplantation [10,23], resection [11,22,25], or transarterial chemoembolization [12]. The role of BMD as an independent predictor of survival is fully consistent with the findings of our study, which analyzed patients with HCC and PVTT.

Patients with PVTT have particularly poor prognoses and are generally assigned to systemic therapy according to the BCLC classification [3,4]. However, not all patients with PVTT have the same prognosis; in clinical reality, individualized treatment approaches according to the BCLC-treatment stage migration concept, such as resection or local ablative therapies, are sometimes used, which necessitates a more accurate assessment of prognosis [3,34]. Furthermore, predicting the response to immunotherapy remains exceptionally challenging, calling for new predictive imaging biomarkers [7]. The prognostic value of BMD in patients with PVTT has not yet been investigated. Only one study has explored the relationship between BMD and vascular invasion in HCC [11]. However, that study had an entirely different scope, as it investigated microvascular invasion in histological specimens after resection. In contrast, our research employed an upfront approach, analyzing cross-sectional imaging to assess macrovascular invasion and BMD with the goal of estimating survival and optimizing treatment stratification.

Previous studies have demonstrated that CT is a suitable method for opportunistic screening of BMD [19–21]. Pickhardt et al. compared BMD measurements obtained from DXA with those from CT colonography performed within two months of the DXA scan, demonstrating a strong correlation between non-enhanced abdominal CT-derived BMD values and those measured by DXA [20]. In clinical practice, many CT scans are performed with contrast enhancement, often without a preceding non-contrast series, restricting BMD evaluation to contrast-enhanced imaging. Despite the presence of intravenous contrast medium, its effect on trabecular bone attenuation is minimal and does not significantly compromise BMD measurements in thoracolumbar vertebrae [21,35]. Following established protocols, we assessed BMD by placing a region of interest in the first lumbar vertebra, which is routinely captured in both abdominal and thoracic CT scans [20,21,31]. In our cohort, the optimal BMD stratification threshold was determined to be 159 HU at both HCC diagnosis and PVTT onset, closely aligning with the widely accepted 160 HU cut-off reported by Pickhardt et al. [21]. This consistency reinforces the validity of our methodology and highlights the representativeness of our patient population.

This study has several limitations. Although our retrospective analysis provides compelling evidence that low BMD is an adverse prognostic factor in patients with HCC and PVTT, the non-randomized design limits causal inference and precludes definitive therapeutic recommendations. Prospective studies are needed to validate these findings and to evaluate whether integrating BMD into established prognostic models—alongside clinical and imaging-based parameters—can enhance risk stratification and enable more individualized outcome prediction in this high-risk population. Given the marked heterogeneity in outcomes among patients with advanced-stage HCC, it is plausible that a subset may benefit from tailored supportive or multimodal therapeutic strategies. This possibility, however, requires confirmation through well-designed prospective trials. Furthermore, the study did not assess the potential survival impact of bone-directed therapies in patients with low BMD. While interventions aimed at preserving or improving bone health may offer clinical benefit, evidence directly linking such strategies to improved survival in this population are still lacking. Importantly, reduced BMD in patients with HCC is often multifactorial. Many—such as those in our cohort—have underlying cirrhosis, which is associated with systemic metabolic dysregulation, including hormonal imbalance, chronic inflammation, malnutrition, and sarcopenia [13–15]. Impaired physical activity due to poor performance status may further exacerbate bone loss. In this context, low BMD should not be viewed solely as a skeletal abnormality, but rather as a surrogate marker of overall physical decline [10,14,24]. Whether bone-targeted pharmacologic interventions can attenuate this decline or improve oncologic outcomes remains an open and clinically relevant question. Moreover, BMD was manually assessed, which, despite being a reliable method, introduces potential interobserver variability. Future advancements in artificial intelligence (AI) are anticipated to facilitate fully automated BMD measurements, enhancing both accuracy and accessibility. AI-driven approaches could further establish BMD as a surrogate marker of overall patient status, enabling more precise and individualized risk stratification. Another limitation is the assessment of BMD based on a single vertebra. However, in cases of bone lesions, compression fractures, or imaging artifacts, measurements were taken from an adjacent vertebra to maintain reliability [12]. This approach provides a rapid and reproducible method suitable for clinical application. Looking ahead, manual BMD assessments will likely be replaced by AI-powered tools applied to all routinely acquired CT scans, fostering the seamless integration into structured radiology reports.

In conclusion, reduced BMD has been identified as an independent prognostic factor for survival in patients with HCC and PVTT. Given its potential role as a surrogate marker of overall patient status, BMD may contribute to more refined survival stratification and inform clinical decision-making. However, prospective validation is required to establish its utility in routine clinical practice.

## Supporting information

S1 Fig Subgroup analysis of patients with synchronous and metachronous PVTT diagnosis. A, Patients with synchronous PVTT and high BMD (≥ 160 HU) had a median OS of 7.5 months, whereas those with low BMD (< 160 HU) had a median OS of 4.2 months (p < 0.001). B, Calculated from the date of initial HCC diagnosis, patients with metachronous PVTT and high BMD had a median OS of 19.7 months, whereas those with low BMD had a median OS of 13.0 months (p = 0.043). C, Calculated from the date of PVTT diagnosis, patients with metachronous PVTT and high BMD had a median OS of 12.3 months, whereas those with low BMD had a median OS of 6.3 months (p < 0.001).
(TIFF)

S2 Fig Distribution of bone mineral density (BMD) among the different HCC growth types at (A) time of first HCC diagnosis and (B) time of PVTT diagnosis. At initial diagnosis of HCC, patients with a diffuse tumor had a median BMD of 142 HU (IQR, 117–161), whereas patients with a nodular tumor had a median BMD of 136 HU (IQR, 113–163, p = 0.32). At PVTT diagnosis, patients with a diffuse tumor had a median BMD of 142 HU (IQR, 116–160), whereas patients with a nodular tumor had a median BMD of 132 HU (IQR, 107–161, p = 0.077).
(TIFF)

S3 Fig Distribution of bone mineral density (BMD) among patients with and without extrahepatic metastatic disease (EHM) at (A) time of first HCC diagnosis and (B) time of PVTT diagnosis. At initial diagnosis of HCC, patients with an EHM had a median BMD of 135 HU (IQR, 111–160), whereas patients without EHM had a median BMD of 140 HU (IQR, 120–164, p = 0.13). At PVTT diagnosis, patients with an EHM had a median BMD of 134 HU (IQR, 107–159), whereas patients without EHM had a median BMD of 133 HU (IQR, 116–158, p = 0.38).
(TIFF)

S1 Table Correlation between bone mineral density (BMD) and surrogates of liver function and tumor burden. Correlation coefficient: > 0.1, weak correlation; > 0.3, moderate correlation; > 0.5, strong correlation. Abbreviations: ALBI, Albumin-Bilirubin; HCC, hepatocellular carcinoma; PVTT, portal vein tumor thrombosis; AFP, alpha-fetoprotein. ^†^Only in patients with nodular growth type.
(DOCX)

## Abbreviations

HCC: Hepatocellular carcinoma
BCLC: Barcelona Clinic Liver Cancer
PVTT: Portal vein tumor thrombosis
BMD: Bone mineral density
OS: Overall survival
DXA: Dual-energy X-ray absorptiometry
CT: Computed tomography
EASL: European Association for the Study of the Liver
AASLD: American Association for the Study of Liver Diseases
HU: Hounsfield unit
IQR: Interquartile range
HRs: Hazard ratios
CIs: Confidence intervals
ALBI: Albumin-Bilirubin
AFP: Alpha-fetoprotein
PS: Performance status
AI: Artificial intelligence.
