# Supplementary figures and images for: Bone mineral density predicts survival in patients with hepatocellular carcinoma and portal vein tumor thrombosis
Source: PLoS One. 2025 Aug 22;20(8):e0330336. doi: 10.1371/journal.pone.0330336 (PMC12373191; doi:10.1371/journal.pone.0330336)

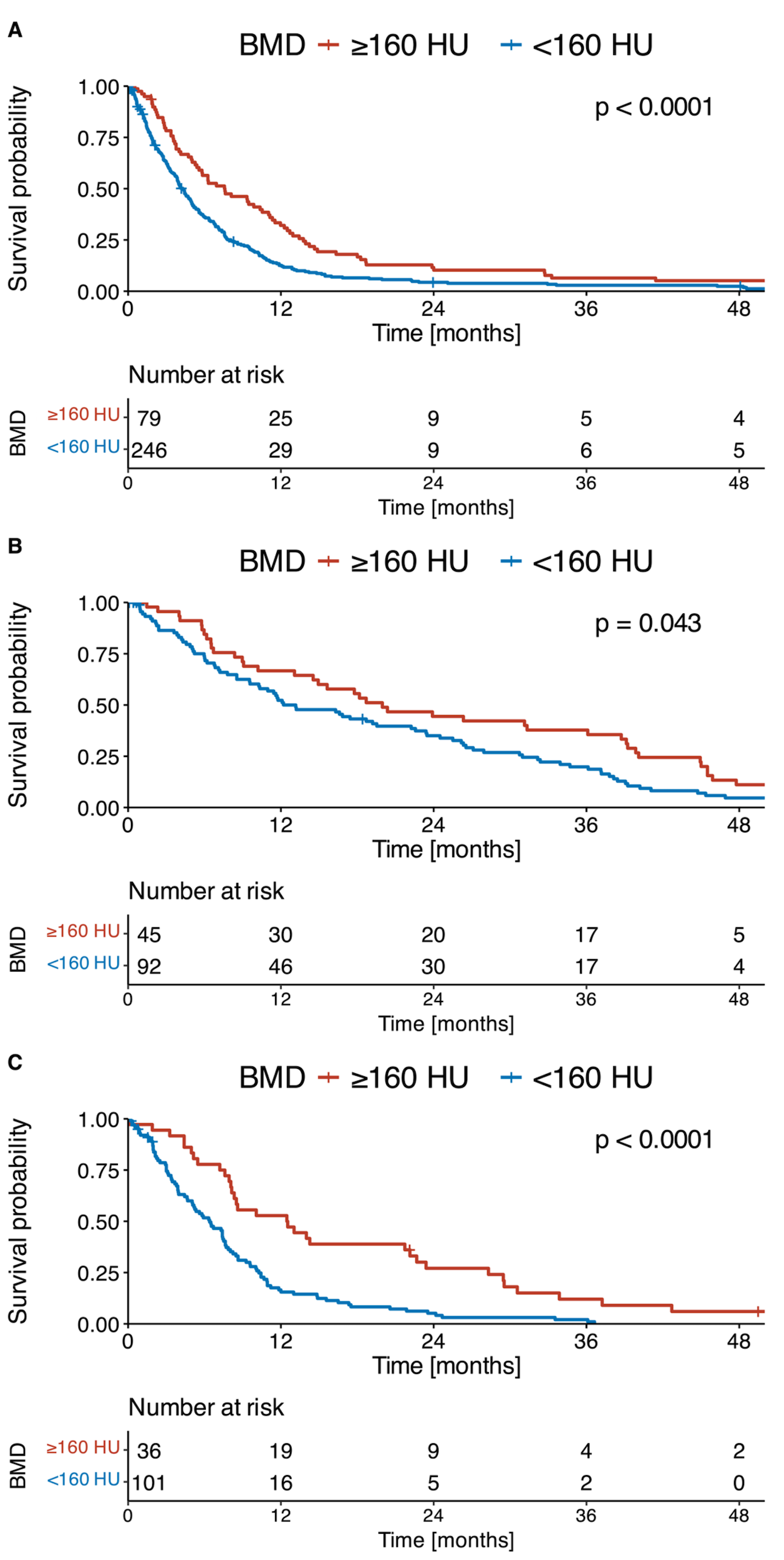

Supplement: S1 Fig — A, Patients with synchronous PVTT and high BMD (≥ 160 HU) had a median OS of 7.5 months, whereas those with low BMD (< 160 HU) had a median OS of 4.2 months (p < 0.001). B, Calculated from the date of initial HCC diagnosis, patients with metachronous PVTT and high BMD had a median OS of 19.7 months, whereas those with low BMD had a median OS of 13.0 months (p = 0.043). C, Calculated from the date of PVTT diagnosis, patients with metachronous PVTT and high BMD had a median OS of 12.3 months, whereas those with low BMD had a median OS of 6.3 months (p < 0.001). (TIFF) [file pone.0330336.s001.tiff]

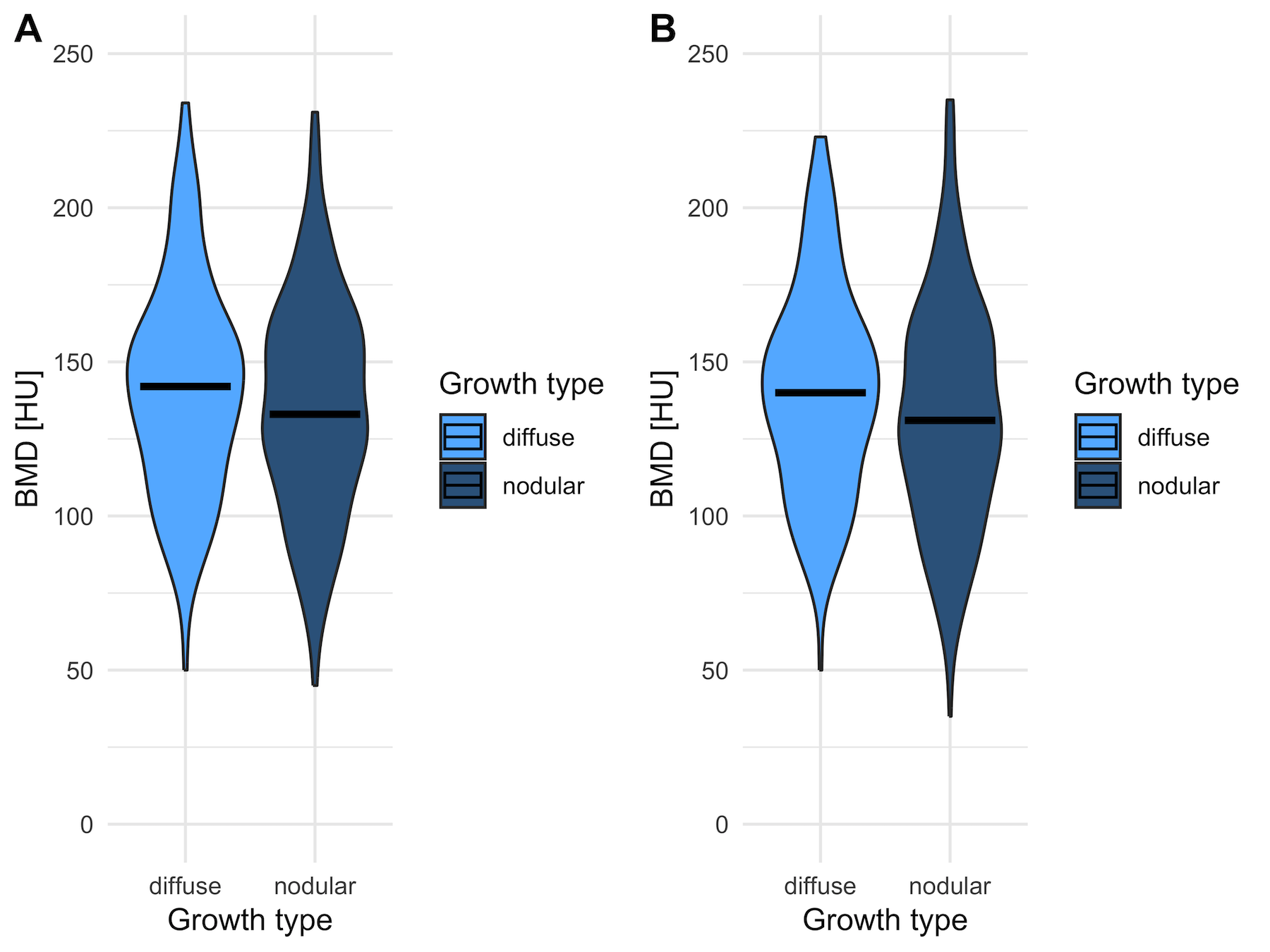

Supplement: S2 Fig — At initial diagnosis of HCC, patients with a diffuse tumor had a median BMD of 142 HU (IQR, 117–161), whereas patients with a nodular tumor had a median BMD of 136 HU (IQR, 113–163, p = 0.32). At PVTT diagnosis, patients with a diffuse tumor had a median BMD of 142 HU (IQR, 116–160), whereas patients with a nodular tumor had a median BMD of 132 HU (IQR, 107–161, p = 0.077). (TIFF) [file pone.0330336.s002.tiff]

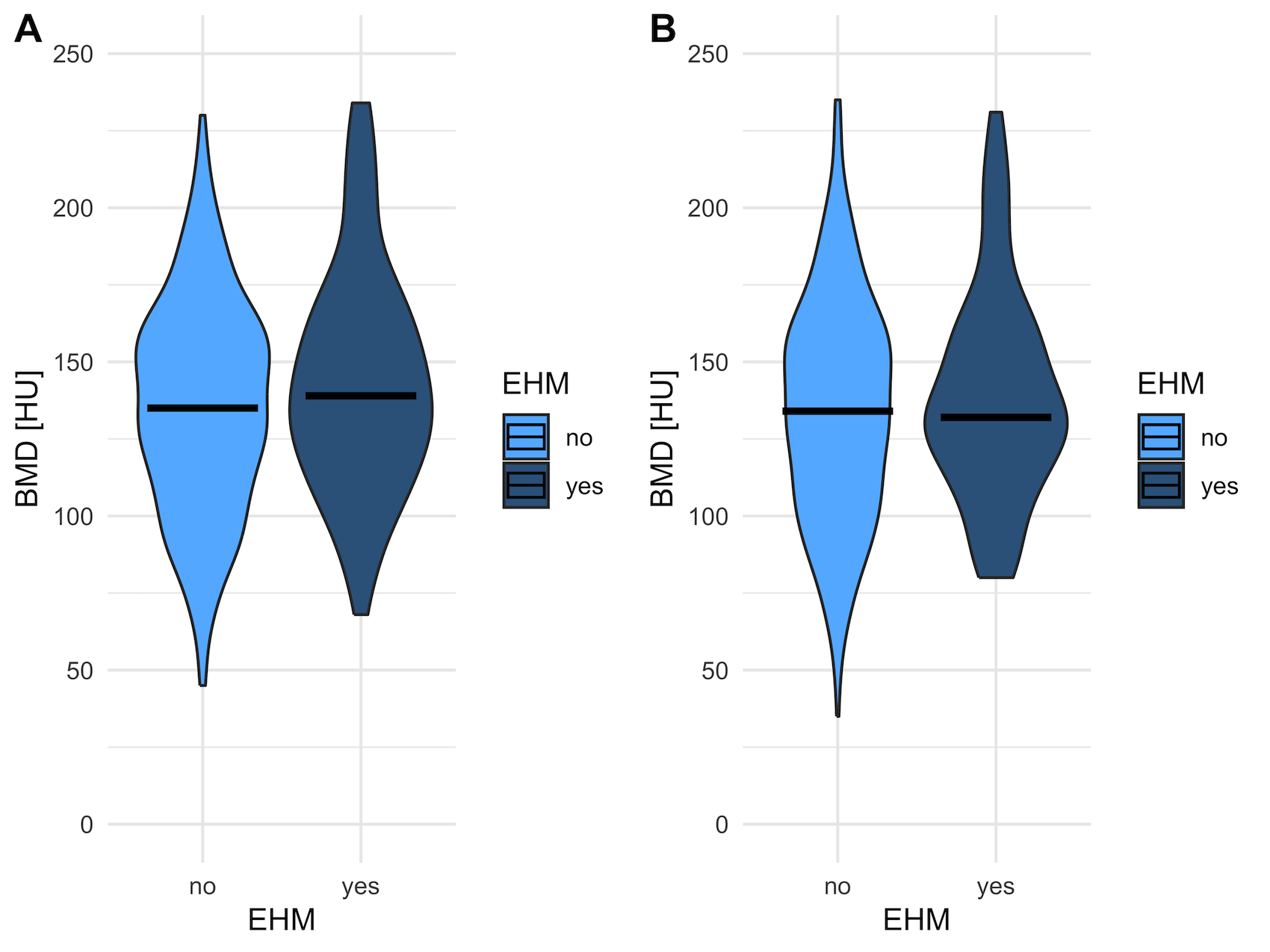

Supplement: S3 Fig — At initial diagnosis of HCC, patients with an EHM had a median BMD of 135 HU (IQR, 111–160), whereas patients without EHM had a median BMD of 140 HU (IQR, 120–164, p = 0.13). At PVTT diagnosis, patients with an EHM had a median BMD of 134 HU (IQR, 107–159), whereas patients without EHM had a median BMD of 133 HU (IQR, 116–158, p = 0.38). (TIFF) [file pone.0330336.s003.tiff]
