# Supplementary material for: Bone mineral density predicts survival in patients with hepatocellular carcinoma and portal vein tumor thrombosis
Source: PLoS One. 2025 Aug 22;20(8):e0330336. doi: 10.1371/journal.pone.0330336 (PMC12373191; doi:10.1371/journal.pone.0330336)
Supplement: S1 Table — Correlation coefficient: > 0.1, weak correlation; > 0.3, moderate correlation; > 0.5, strong correlation. Abbreviations: ALBI, Albumin-Bilirubin; HCC, hepatocellular carcinoma; PVTT, portal vein tumor thrombosis; AFP, alpha-fetoprotein. †Only in patients with nodular growth type. (DOCX) [file pone.0330336.s004.docx]

| **Parameter** | **Correlation coefficient** | **P-value** |
| --- | --- | --- |
| **Liver function** |  |  |
| ALBI grade *– HCC diagnosis* | 0.02 | 0.73 |
| ALBI grade *– PVTT diagnosis* | 0.01 | 0.80 |
| **Tumor burden and tumor marker** |  |  |
| Largest tumor diameter *– HCC diagnosis* | -0.14 | 0.03 |
| Number of tumors *– HCC diagnosis^†^* | 0.01 | 0.88 |
| AFP level *– HCC diagnosis* | 0.03 | 0.59 |
| Largest tumor diameter *– PVTT diagnosis* | -0.12 | 0.07 |
| Number of tumors *– PVTT diagnosis^†^* | 0.01 | 0.93 |
| AFP level *– PVTT diagnosis* | 0.05 | 0.35 |
